# PEEP titration during prone positioning for acute respiratory distress syndrome

**DOI:** 10.1186/s13054-015-1153-9

**Published:** 2015-12-21

**Authors:** Jeremy R. Beitler, Claude Guérin, Louis Ayzac, Jordi Mancebo, Dina M. Bates, Atul Malhotra, Daniel Talmor

**Affiliations:** Division of Pulmonary and Critical Care Medicine, University of California, 200 West Arbor Drive, San Diego, CA 92103 USA; Service de Réanimation Médicale, Hôpital de la Croix-Rousse, Université de Lyon, 103 Grande Rue de la Croix-Rousse, 69004 Lyon, France; INSERM 955 Eq.13, Créteil, France; Centre de Coordination et de Lutte contre les Infections Nosocomiales Sud-Est, Hôpital Henri Gabrielle, 20 Routes de Vourles, 69230 Saint-Genis-Laval, France; Servei de Medicina Intensiva, Hospital de Sant Pau, Avinguda Sant Antoni Maria Claret 167, 08025 Barcelona, Spain; Department of Anesthesia, Critical Care, and Pain Medicine, Beth Israel Deaconess Medical Center, 330 Brookline Ave, Boston, MA 02215 USA

## Abstract

No major trial evaluating prone positioning for acute respiratory distress syndrome (ARDS) has incorporated a high-positive end-expiratory pressure (high-PEEP) strategy despite complementary physiological rationales. We evaluated generalizability of three recent proning trials to patients receiving a high-PEEP strategy. All trials employed a relatively low-PEEP strategy. After protocol ventilator settings were initiated and the patient was positioned per treatment assignment, post-intervention PEEP was not more than 5 cm H_2_O in 16.7 % and not more than 10 cm H_2_O in 66.0 % of patients. Post-intervention PEEP would have been nearly twice the set PEEP had a high-PEEP strategy been employed. Use of either proning or high-PEEP likely improves survival in moderate-severe ARDS; the role for both concomitantly remains unknown.

## Introduction

Prone positioning has been used since the 1970s to improve oxygenation in patients with acute respiratory failure [1]. Several important physiological changes occur with proning: recruitment of dependent lung regions [2, 3], increased ventilation-perfusion matching [4], optimized chest wall mechanics [5], decreased pleural pressure gradient from non-dependent to dependent regions [6, 7], and augmented drainage of tracheobronchial secretions [8].

Given these effects, proning would seem likely to benefit patients with acute respiratory distress syndrome (ARDS). Indeed, the recent PROSEVA (Proning Severe ARDS Patients) multicenter randomized trial demonstrated significantly improved survival with proning for moderate-severe ARDS [9]. This trial built on cumulative experience from the prior decade of indeterminate trials [10–15], which served to refine the target population, intervention strategy, and co-interventions. Key features of PROSEVA that collectively may explain the observed benefit compared with prior trials include enrollment of patients only with more severe ARDS (partial pressure of oxygen in arterial blood/fraction of inspired oxygen (PaO_2_/FiO_2_) of less than 150), protocolized low tidal volume ventilation, near-ubiquitous use of continuous neuromuscular blockade, intervening early in the ARDS course, and increased duration of prone positioning [16].

High positive end-expiratory pressure (PEEP) has been advocated as part of an “open lung” approach by citing a rationale similar to that of proning—improving lung homogeneity to minimize ventilator-induced lung injury [16]. Although no large clinical trial to date has demonstrated definitively a mortality benefit with high PEEP [17–19], a meta-analysis of three such trials found significantly increased survival and ventilator-free days with high PEEP for moderate-severe ARDS [20]. As a result, several recently completed and ongoing multicenter clinical trials of ARDS have incorporated a high-PEEP strategy [21–23], although this practice is not universal [24–29].

No major randomized trial of prone versus supine positioning has incorporated a high-PEEP strategy for either study arm [16]. Thus, it is unclear to what extent patients managed with a high-PEEP strategy may gain benefit from prone positioning and vice versa. High PEEP with supine positioning may offer benefit comparable to that of low PEEP with prone positioning [16]; additionally, the combination of high PEEP and proning may be synergistic [30]. To explore this potential limitation of the existing literature further, we conducted a post-hoc analysis of data from recent clinical trials on prone positioning to examine in detail the PEEP titration strategies used and to determine whether PEEP would have been meaningfully different had a high-PEEP strategy been employed.

## Methods

Corresponding authors from all clinical trials identified in a recent meta-analysis on prone positioning for ARDS [16] were contacted. De-identified patient-level data from three of seven trials were ultimately provided [9, 11, 13], including the two largest proning trials to date [9, 11]. One included trial enrolled patients with acute hypoxemic respiratory failure (PaO_2_/FiO_2_ of not more than 300) who had an anticipated duration of mechanical ventilation of more than 48 hours, of which a subgroup met criteria for ARDS [11]. The other two trials enrolled only patients with moderate-severe ARDS, requiring PaO_2_/FiO_2_ of not more than 200 [13] and not more than 150 [9].

### PEEP titration strategy

Clinical trial protocols were reviewed for PEEP titration strategy. Individual patient PEEP settings were evaluated at two predefined thresholds—not more than 5 cm H_2_O and not more than 10 cm H_2_O—at each of two time points: baseline and post-intervention. Baseline PEEP refers to values documented after study enrollment but prior to any study intervention, thus reflecting usual care received by patients before the study. Post-intervention PEEP refers to the first documented values after being placed on protocol ventilator settings and positioned either prone or supine per treatment assignment. To avoid risk of informative censoring owing to death or liberation from mechanical ventilation, later time points were not evaluated.

### Calculation of expected high PEEP

We also considered what PEEP would have been in the proning trials had a high-PEEP protocol been used. This hypothetical PEEP was calculated for each patient by identifying the PEEP level from PEEP-FiO_2_ titration tables that corresponded to each individual’s actual preset FiO_2_. PEEP-FiO_2_ titration tables were obtained from the ALVEOLI (ARDS Network Assessment of Low tidal Volume and elevated End-expiratory volume to Obviate Lung Injury Trial) and LOVS (Lung Open Ventilation Study) protocols, two multicenter trials that compared high- versus low-PEEP strategies [17, 18]. When the titration protocol permitted more than one PEEP setting for a given FiO_2_, the lower PEEP level was reported, biasing toward smaller differences between set and hypothetical high-PEEP values.

### Statistical analysis

Results are reported as mean ± standard deviation or number (percentage) as appropriate. Differences between set PEEP and expected high PEEP were compared by using the paired *t* test. Missing data were thought to occur at random given the timing of our analyses immediately after trial enrollment; thus, patients with missing data were excluded from analysis. Statistical significance was determined by using a two-sided alpha of 0.05.

## Results

Patient-level data from 1365 of 1393 patients, spanning three multicenter randomized trials of prone versus supine positioning, were available and included. The study protocol PEEP titration strategy differed among included trials. In one trial, PEEP was titrated according to non-protocolized usual care [11]. In another [13], PEEP was targeted to 10–15 cm H_2_O, although this upper limit could be exceeded in cases of refractory hypoxemia despite an FiO_2_ of 1.0. In PROSEVA, PEEP was titrated according to the low-PEEP arm of the US National Heart, Lung, and Blood Institute ARDS Network ALVEOLI trial [17].

Baseline PEEP was not more than 5 cm H_2_O in 21.8 % of all participants in pooled analysis and in a similar proportion (18.9 %) in PROSEVA specifically (Table 1). After patients were placed on protocol ventilator settings and positioned prone or supine per treatment assignment, post-intervention PEEP was not more than 5 cm H_2_O in 16.7 % of patients and not more than 10 cm H_2_O in two thirds of patients (66.0 %) in pooled analysis. In regard to PROSEVA, which restricted enrollment to patients with PaO_2_/FiO_2_ of less than 150, post-intervention PEEP was not more than 5 cm H_2_O in 2.1 % of patients and not more than 10 cm H_2_O in half of patients (48.7 %). Neither mean post-intervention PEEP nor the proportion of patients with post-intervention PEEP of not more than 10 cm H_2_O differed significantly by treatment assignment in any of the trials.

**Table 1:** Oxygenation and positive end-expiratory pressure measurements before and after study intervention

	Guerin et al. [11] (2004)	Mancebo et al. [13] (2006)	Guerin et al. [9] (2013)	All studies combined
Baseline				
FiO_2_	66 ± 21	82 ± 20	79 ± 16	71 ± 20
PaO_2_/FiO_2_	152 ± 59	145 ± 84	104 ± 25	136 ± 58
PEEP, cm H_2_O	8 ± 3	12 ± 2	10 ± 3	9 ± 4
PEEP ≤5 cm H_2_O, %	27.2 %	0 %	18.9 %	21.8 %
PEEP ≤10 cm H_2_O, %	85.8 %	29.4 %	62.6 %	72.7 %
Post-intervention				
FiO_2_	59 ± 19	75 ± 20	73 ± 16	65 ± 20
PaO_2_/FiO_2_	179 ± 77	173 ± 88	153 ± 72	170 ± 77
PEEP, cm H_2_O	8 ± 3	13 ± 2	12 ± 3	10 ± 4
PEEP ≤5 cm H_2_O, %	28.0 %	0 %	2.1 %	16.7 %
PEEP ≤10 cm H_2_O, %	83.3 %	24.1 %	48.7 %	66.0 %

Data are presented as mean ± standard deviation or percentage of study participants

*FiO*_*2*_ fraction of inspired oxygen, *PaO*_*2*_ partial pressure of oxygen in arterial blood, *PEEP* positive end-expiratory pressure

**Fig. 1:**
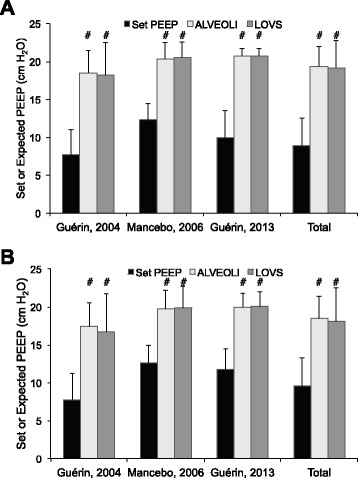
Comparison of set PEEP used in proning clinical trials and hypothetical PEEP that would have been required under the ALVEOLI or LOVS high-PEEP protocols. Expected PEEP under high-PEEP protocols was calculated by using individual patient FiO_2_ values. When the study protocol permitted multiple PEEP levels for a given FiO_2_, the lowest PEEP value was chosen to bias results toward smaller difference between set and expected PEEP. **a** Baseline PEEP values prior to study intervention. **b** First post-intervention PEEP values. Column height and error bars represent mean and standard deviation, respectively. ^#^Significantly different than set PEEP at *P* < 0.05. *ALVEOLI* ARDS Network Assessment of Low tidal Volume and elevated End-expiratory volume to Obviate Lung Injury Trial, *FiO*_*2*_ fraction of inspired oxygen, *LOVS* Lung Open Ventilation Study, *PEEP* positive end-expiratory pressure

In regard to hypothetical high-PEEP values for all trials, mean baseline and post-intervention PEEP both would have doubled had either the ALVEOLI or LOVS high-PEEP strategies been used (*P* < 0.001 for both comparisons with set PEEP) (Fig. 1). In the PROSEVA trial, mean post-intervention set PEEP was 12 ± 3 cm H_2_O, whereas mean expected PEEP under the ALVEOLI and LOVS protocols was 20 ± 2 cm H_2_O (*P* < 0.001 for both comparisons with set PEEP).

## Discussion

The present analysis highlights the paucity of data on concomitant high PEEP with prone positioning. Given the purported overlapping benefits to regional mechanics afforded by both interventions, these data raise legitimate concern about generalizability of existing clinical trial data on prone positioning to patients receiving a high-PEEP strategy. Our findings highlight the need for additional research comparing prone positioning, high PEEP, and both in combination for treatment of moderate-severe ARDS.

The mechanisms by which high PEEP may protect against lung injury are similar to those associated with proning. Both therapies promote more homogenous lung recruitment, minimizing local shear stress; reduce cyclic opening/collapsing of potentially recruitable lung units during tidal ventilation; and improve ventilation-perfusion matching. Indeed, preclinical models have demonstrated that both proning and PEEP mitigate ventilator-induced lung injury [31–33]. Clinically, the PROSEVA trial demonstrated definitively better survival from moderate-severe ARDS with prone compared with supine positioning when managed with a low-PEEP strategy [9]. A similar survival benefit has been suggested with a high-PEEP supine strategy, compared with a low-PEEP supine strategy, for moderate-severe ARDS [20]. No multicenter randomized trial testing a high- versus low-PEEP strategy has demonstrated definitively a survival benefit nor enrolled exclusively patients with moderate-severe ARDS (PaO_2_/FiO_2_ of not more than 200). However, a meta-analysis of the three largest trials found improved survival with high PEEP in the subgroup of patients with PaO_2_/FiO_2_ of not more than 200 [20], comparable to the population enrolled in PROSEVA. Thus, the overlapping physiological and clinical effects of proning and high PEEP raise doubt as to whether a proning low-PEEP strategy yields a survival advantage compared with a high-PEEP supine strategy; further studies are needed to address this question.

Moreover, whether concomitant provision of high PEEP and proning offers additional clinical benefit over either alone is unknown. In patients with diffuse infiltrates on chest computed tomography (CT), combination therapy improves oxygenation and reduces intrapulmonary shunt compared with either monotherapy [34]. Prone positioning may reduce chest wall compliance [35], potentially necessitating higher PEEP to offset these changes. A recent CT study of 24 patients with ARDS found that cyclic atelectasis decreased only when higher PEEP (15 versus 5 cm H_2_O) and prone positioning were applied together. Whereas each therapy in isolation promoted lung recruitment, simultaneous proning and higher PEEP also mitigated regional hyperinflation observed with higher PEEP during supine positioning. An individually titrated high-PEEP strategy in combination with proning has not been studied rigorously to date. The ideal combination therapy may require adjusting PEEP after each repositioning to account for changes in chest wall and lung mechanics in the prone versus supine position.

Importantly, the optimum approach to setting PEEP remains undefined. In this study, high PEEP was estimated by using PEEP-FiO_2_ titration tables of the ALVEOLI and LOVS trials. Such an “open lung” strategy was shown recently to achieve higher levels of PEEP in patients with more lung recruitability as measured by CT scan, whereas other methods—ExPress, stress index, and esophageal manometry—had no association with lung recruitability [36]. Most major clinical trials to date have titrated PEEP according to severity of oxygenation impairment by using an arbitrary PEEP-FiO_2_ table with comparably lower PEEP [17, 18]. However, mechanical insults and resultant biotrauma appear to be the primary drivers of ventilator-induced lung injury [37]. Thus, we believe that the optimum approach involves titrating PEEP to minimize mechanical lung injury. Several mechanics-based approaches have been proposed: setting PEEP above the lower inflection point of the static pressure-volume curve [38, 39], according to pleural pressure (estimated by esophageal manometry) to achieve a non-negative end-expiratory transpulmonary pressure (airway minus pleural pressure) [23, 40], and according to highest respiratory system compliance [41, 42] or lowest driving pressure (airway end-inspiratory plateau pressure minus PEEP) [43], among others. Mechanics-based PEEP strategies yield different PEEP selections than high PEEP-FiO_2_ tables [36]. To maximize synergy with proning, a mechanics-based approach to PEEP adjustment may be necessary to further improve lung homogeneity, avoid overdistension or hemodynamic compromise, and prevent cyclic opening/collapse of potentially recruitable lung units [44]. Changes in chest wall compliance (decreased when prone) and aerated lung volume (increased when prone) following repositioning highlight the apparent need to adjust PEEP after each position change to optimize mechanics for lung injury prevention.

Some important limitations to the present study are of note. First, although no included trial incorporated a high-PEEP titration strategy, the particular PEEP strategy used differs for each included study. Only the PROSEVA trial used a PEEP-FiO_2_ titration table for all patients, identical to the low-PEEP strategy employed in the ALVEOLI trial. Second, our calculation of expected high PEEP may overestimate what the true PEEP level would have been had a high PEEP-FiO_2_ table been used. Expected PEEP was calculated by using a PEEP-FiO_2_ table, although a mechanics-based approach is preferred in our view. Increasing PEEP often improves oxygenation, permitting a reduction in FiO_2_ while maintaining PaO_2_ or SpO_2_ (blood oxygen saturation measured by pulse oximetry) goals. High PEEP occasionally also could contribute to hemodynamic instability, particularly in patients with high lung compliance in whom overdistension is likelier. These limitations, which would lower PEEP, are offset in part by our reporting the lowest expected PEEP level allowed whenever the high-PEEP titration protocol permitted more than one PEEP setting for a given FiO_2_. Third, it is unknown whether a clinically meaningful interaction between PEEP and prone positioning exists, as simultaneous provision of these therapies has never been studied in a clinical trial powered for patient-centered endpoints. Finally, it is unclear whether high PEEP would have altered clinical outcomes in either the prone or supine study arms, although existing data suggest a benefit with supine positioning in moderate-severe ARDS [20].

## Conclusions

Existing clinical trial data on proning may not be generalizable to patients receiving high PEEP. Current evidence supports the routine use of either proning or high PEEP in moderate-severe ARDS. A prudent approach may be to tailor the choice of strategy to individual patient safety factors, relative contraindications, and staff training/experience. Although concomitant use of proning and high PEEP has never been studied in a clinical trial with patient-centered endpoints, effects may be synergistic. Future clinical trials are needed to compare efficacy and safety of proning, high-PEEP, and both in combination for moderate-severe ARDS.

## Abbreviations

ALVEOLI: ARDS Network Assessment of Low tidal Volume and elevated End-expiratory volume to Obviate Lung Injury Trial
ARDS: acute respiratory distress syndrome
CT: computed tomography
FiO_2_: fraction of inspired oxygen
LOVS: Lung Open Ventilation Study
PaO_2_: partial pressure of oxygen in arterial blood
PEEP: positive end-expiratory pressure
PROSEVA: Proning Severe ARDS Patients
